# Suicide Risk Screening and Assessment before and after the COVID-19 Pandemic in New Inmates

**DOI:** 10.3390/healthcare12010100

**Published:** 2024-01-02

**Authors:** Carmen Santoriello, Carmela De Rosa, Chiara Rufo, Francesca Romano, Gaetana Termoli, Giuseppina Fiorillo, Ludovica Caprio, Monica Vitolo, Antonio Maria Pagano

**Affiliations:** Unità Operativa Semplice Dipartimentale U.O.S.D. Department of Adults and Minors Healthcare, Criminal Area, Local Health Authority of Salerno, 84132 Salerno, Italy

**Keywords:** suicide prevention, prisoners, health, psychosocial distress, suicidal behavior

## Abstract

(1) Background: Suicide is the main cause of death in Italian prisons. The largest number of inmates who killed themselves was recorded during three years of the COVID-19 pandemic. This study aimed to explore psychosocial risk factors for suicide among inmates incarcerated before and after the onset of COVID-19. (2) Methods: At prison reception, inmates underwent clinical interviews and were assessed using the Blaauw Scale and Suicide Assessment Scale. Psychological distress, measured by the Symptom Checklist-90-R, was compared between inmates admitted before and after COVID-19. Regression analyses were run to examine psychosocial vulnerabilities associated with suicidal intent in newly incarcerated individuals at risk of suicide. (3) Results: Among the 2098 newly admitted inmates (93.7% male) aged 18 to 87 years (M = 39.93; SD = 12.04), 1347 met the criteria for suicide risk, and 98 exhibited high suicidal intent. Inmates who entered prison after the onset of COVID-19 were older and had fewer social relationships. They had a higher prevalence of recidivism and substance abuse, along with elevated levels of psychological distress. An increase in perceived loss of control, anergia, obsessive-compulsive symptoms, phobic anxiety, and paranoid ideation emerged as the factors most strongly associated with high suicidal intent. (4) Conclusions: These findings support the value of psychosocial screening in promptly identifying inmates at risk of suicide, enabling the implementation of targeted, multi-professional interventions. Future research should replicate these results, with a focus on longitudinal studies that monitor the same inmates throughout their incarceration period.

## 1. Introduction

Suicide is self-directed, injurious behavior with the intent to die. A suicide attempt is when self-injurious behavior, planned and acted upon to achieve death, results in a non-fatal outcome [1,2,3]. Suicidal intent, defined as the intensity of the person’s wish to terminate his or her life, has been found to predict completed suicide [4,5,6,7,8,9]. Self-injurious behavior and suicide attempts are more common among inmates than non-inmates [10,11,12,13]. In 2007, the International Association for Suicide Prevention established the New Task Force on Suicide in Prisons, which updated the ‘Preventing Suicide’ guide [14], originally published by the World Health Organization’s Department of Mental Health.

Konrad et al. [14] emphasized the importance of identifying and treating inmates’ vulnerability to suicidal behavior throughout their incarceration, with particular attention to the prison reception period [15,16,17,18,19,20,21]. The stress-vulnerability model describes suicidal behavior as a multidimensional process that evolves through the interaction of individual and environmental variables [22,23]. Inmates’ psychosocial vulnerabilities, such as social alienation, cognitive distortions, and deficient adaptive resources, along with poor coping skills to handle distress and prison stressors (e.g., uncertainty, separation from family, forced cohabitation with other inmates), may trigger suicidal ideation [22,23,24]. 

The COVID-19 pandemic has worsened distress and the pain of imprisonment [25,26,27,28]. In a Swiss prison, even with reduced overcrowding, there was a 57% increase in suicide attempt risk during the pandemic period [29]. A recent systematic review on the impact of COVID-19 on the health of individuals in detention [30] highlighted that isolation worsened inmates’ psychological pain, frustration, and stress [31,32,33], leading to increased anxiety, self-harm behavior, and suicidal ideation [34,35]. According to the latest data from the WHO (Suicide Worldwide in 2019), Italy had a suicide rate of 0.67 per 10,000 people [36]. However, according to Antigone’s Annual Reports on Detention Conditions, the suicide rate in Italian prisons was 8.7 per 10,000 incarcerated individuals in 2019, 11 in 2020, and reached a peak of 15.4 in 2022, the highest ever recorded [37,38,39]. Initially, a substantial consistency of evidence at both regional and national levels is likely.

In recent years, the suicide death trend in the penitentiary institutions of the Campania Region (Italy) saw an increase in the suicide rate, rising from 0.8 per 1000 inmates in 2019 to 1.4 in 2020 and to 1.04 in both 2021 and 2022. [40]. Despite the fact that suicide deaths in Italian prisons are currently 23 times higher than suicides in the general population, and the largest number was recorded during years of coronavirus disease [39], few studies have focused on psychosocial risk factors for suicidal behavior in newly admitted inmates [13,41,42,43] incarcerated before and after the onset of COVID-19. 

The multidisciplinary team of the U.O.S.D. Department of Adults and Minors Healthcare, Criminal Area, Local Health Authority (ASL) of Salerno, operating in three different penitentiary facilities, has implemented screening protocols for preventing suicidal and self-injurious behavior, as well as operational protocols for COVID-19 infection prevention and screening in prisons [44]. The primary objective of this retrospective study was to investigate psychosocial vulnerabilities associated with suicidal intent in inmates upon their reception in prison. First, we aimed to characterize the socio-demographic, clinical, and legal profiles of a large sample of newly admitted inmates. Then, we tested two hypotheses: 

(1) The psychological distress symptoms of new inmates with suicide risk and high suicidal intent who entered prison before the onset of COVID-19 significantly differ from those of new inmates with suicide risk and high suicidal intent who entered prison after the pandemic’s onset.

(2) Whether inmates’ psychosocial vulnerabilities can effectively differentiate between high and low suicidal intent upon prison reception, the Suicide Risk Screening and Assessment (SRSA) protocol proposed in this study may prove useful in early detection of inmates at an increased risk of suicide attempts, allowing for the prompt implementation of targeted interventions to prevent self-injurious behaviors.

## 2. Materials and Methods 

### 2.1. Participants

This retrospective study included blinded data from 2098 new inmates collected between January 2017 and September 2023 at the Southern Italy Penitentiary “A. Caputo” in Salerno. All new inmates were enrolled after providing written informed consent. They were interviewed by a clinical psychologist within 24 h of entering prison to assess their suicide risk. After the initial screening, inmates at risk of suicide underwent a second clinical interview to identify symptoms of psychological distress and suicidal intent. 

### 2.2. Procedure

The Suicide Risk Screening and Assessment (SRSA) protocol encompassed the assessment of socio-demographic, clinical, and legal characteristics. A clinical psychologist administered a demographic questionnaire to new inmates, recording data including gender, age, nationality, education, marital status, occupation, social network, previous self-harm and suicide attempts, substance addiction, and offending behavior.

#### 2.2.1. Self-Injurious Behavior and Suicide Risk

The Blaauw Scale is a suicide risk screening tool for Penal Institutions [45]. It consists of eight questions, each assigned a weight: 1. Age 40 or older; 2. No fixed address or residence shortly before confinement; 3. One prior confinement; 4. History of multiple cases of hard drug abuse; 5. History of treatment for mental symptoms; 6. Diagnosis of a psychotic disorder or other DSM-IV Axis-1 disorder in the past five years; 7. Previous suicide attempts or self-destructive behaviors; 8. Suicidal utterances or suicide attempts during the admission interview or other specified situations. As reported by the authors, the Scale accurately identified 95% of inmates at high-risk of suicide using a cut-off of 24. Inmates were interviewed by a psychologist in either Italian or English. Those scoring 24 points or more on the Blaauw Scale were classified as having suicide risk (Blaauw+) and underwent further assessment for psychological distress and suicidal intent symptoms. 

#### 2.2.2. Psychological Distress

The Symptom Checklist 90-Revised (SCL-90-R) is a self-report assessment tool that measures the severity of psychological distress symptoms experienced in the last 7 days [46,47]. It comprises 90 symptom descriptions, with individuals rating their severity on a scale from 0 (Not at all) to 4 (Extremely). The SCL-90-R assesses symptoms across nine dimensions: Somatization, Obsessive-Compulsive, Interpersonal Sensitivity, Depression, Anxiety, Hostility, Phobic Anxiety, Paranoid Ideation, and Psychoticism. Additionally, the SCL-90-R includes three global indices of psychological distress. The Global Severity Index (GSI) is the primary indicator of an individual’s current level of psychological distress. Positive Symptom Total (PST) measures the breadth of symptoms. The Positive Symptom Distress Index (PSDI) quantifies the intensity of endorsed symptoms. SCL-90-R scores are standardized, with a mean of 50 and a standard deviation of 10. Gender-specific norms are available in the scoring kit for matching T-scores with percentiles. The Italian translation and validation [47] showed Cronbach’s α values ranging from 0.68 to 0.87 for the nine dimensions and 0.97 for the GSI score.

#### 2.2.3. Suicidal Intent

The Suicide Assessment Scale (SUAS) is a semi-structured clinical interview designed to independently assess suicidal intent symptoms and intensity [48]. The SUAS Scale comprises 20 items, each evaluated on a 5-point scale, resulting in a potential range of 0 to 80. These items are categorized into five domains: (1) Affect, covering Sadness, Hostility, Anxiety, Low Self-esteem, and Hopelessness; (2) Control and coping, including Resourcefulness, Perceived loss of control, Impulsivity, and Poor frustration tolerance; (3) Emotional reactivity, which assesses Hypersensitivity, Emotional withdrawal, and Lack of emotional contact; (4) Bodily states, encompassing Anergia, Tension, and Somatic concerns; (5) Suicidal thoughts and behavior, evaluating Suicidal thoughts, Purpose of suicide, Wish to die, Lack of reason for living, and Suicidal actions. A SUAS cut-off point of 39 yielded a sensitivity of 75% and a specificity of 86.3%. Inmates scoring 40 points or more on the SUAS Scale were classified as having high suicidal intent intensity (SUAS+). 

### 2.3. Data Analysis 

The sample of 2098 new inmates was divided into two groups based on their admission dates: pre-COVID-19 (January 2017 to February 2020) and post-COVID-19 (March 2020 to September 2023). These two groups were further divided into subgroups based on Blaauw Scale-total scores (Blaauw+ for suicide risk) and Suicide Assessment Scale total scores (SUAS+ for high suicidal intent). The Shapiro–Wilk test were used to check for normality of the data distribution, and homogeneity of variance was estimated using Levene’s test. Non-parametric analyses were used when the assumptions of homogeneity of variance or normality were violated (*p* < 0.05). A first analysis was carried out to assess the socio-demographic, clinical, and legal characteristics of the groups. Categorical variable comparisons were performed using Fisher’s exact test or Pearson’s Chi-square test, as appropriate, and the effect sizes (Cramer’s *V*) were calculated. Continuous variables (i.e., SUAS and SCL-90-R scores) were compared between-groups and subgroups using the Student’s *t*-test or Mann–Whitney U test for independent groups. Binary logistic regression analyses were run to assess the impact of psychological distress symptoms on high suicidal intent (SUAS+), while controlling for the Global Severity Index (GSI > 65 T-scores). Additional regression analyses were conducted using psychological distress symptoms (below or above the clinical attention threshold) as outcomes, with socio-demographic, clinical, and legal variables as predictors, adjusting for SUAS+. Statistical analyses were performed using IBM SPSS Statistics software (version 21.0), adopting an alpha error rate of 0.05 (two-tailed) and a statistical power of 95%.

### 2.4. Ethical Issues 

This study was conducted in accordance with the Declaration of Helsinki. All studies regarding the Italian Penitentiary System have been approved by the Ethics Committee of the University of Rome “Tor Vergata” [Registro sperimentazioni 73/05].

## 3. Results

### 3.1. Socio-Demographic Clinical and Legal Characteristics of New Inmates 

The sample comprised 2098 newly admitted inmates (93.7% male; mean age 39.93 ± 12.04 years; range 18–87). The majority (88%) were Italian, and 81% had less than 12 years of education. Approximately 54% were employed at the time of imprisonment. Regarding marital status, 51% were married or cohabiting, 36% were single, and 13% were separated, divorced, or widowed. In terms of their social net, 53% relied on extended family, 30% lived with their family of origin, and 17% had no family support. A significant portion of the sample reported no previous self-harm (91%) or suicide attempts (92%). Substance abuse was reported by 80% of the inmates, and 63% had prior convictions, making them recidivists. The offenses varied, with 56% of inmates having committed drug-related crimes, 41% having committed embezzlement crimes, 23% being involved in criminal conspiracy, and 12% having committed violence against people. 64% of the sample met the criteria for suicide risk.

### 3.2. Comparisons of Variables in Pre-and Post-COVID-19 Groups and Subgroups

The pre-COVID-19 group included 1043 inmates (94.2% male; mean age = 39.34 ± 11.64 years; range 18–76), of whom 70% had suicide risk (Blaauw+: n = 726; 93.1% male; mean age = 41.08 ± 11.24 years). The post-COVID-19 group consisted of 1055 inmates (93.3% male; mean age = 40.57 ± 12.32 years; range 18–87); 59% had suicide risk (Blaauw+: n = 621; 94.5% male; mean age = 43.01 ± 12.01 years). A descriptive summary of other socio-demographic, clinical, and legal characteristics of the two groups and subgroups appears in Table 1. Significant differences were observed between groups in terms of age range, nationality, education, occupation, social net, and offending behavior variables. Within the subgroups of inmates with suicide risk (Blaauw+), significant differences were found in specific age ranges, marital status, social network, substance addiction, crime types, and offending behavior. Compared to the pre-COVID-19 period, the post-COVID-19 period witnessed a significantly higher proportion of inmates at risk of suicide who were older than 60 (8.9% vs. 5.4%), single (35.8% vs. 30.4%), separated or widowed (15.8% vs. 11.4%), lacking a family (19.3% vs. 10.3%), substance abusers (86.3% vs. 78.1%), repeat offenders (77.1% vs. 43.8%), and involved in various types of crime, notably embezzlement (47.2% vs. 38.4%), criminal conspiracy (27.7% vs. 19.1%), and violence against persons (14% vs. 8.8%).

### 3.3. Comparisons of Psychological Distress and Suicidal Intent Symptoms in New Inmates

Since the data were not normally distributed (Shapiro–Wilk test, *p* < 0.05) and the variances between groups were unequal (Levene’s test, *p* < 0.05), non-parametric analyses were conducted. Out of the 1043 inmates admitted before COVID-19, approximately 70% had suicide risk (Blaauw+; N = 726), with 12% exhibiting high suicidal intent (SUAS+; N = 85; 93% male; mean age = 39.36 ± 10.85 years). In contrast, among the 1055 inmates admitted after COVID-19, around 59% had suicide risk (Blaauw+; N = 621), but only 2% had high suicidal intent (SUAS+; N = 13; 85% male; mean age = 41.51 ± 13.23 years). Statistical comparisons conducted using the Mann–Whitney U test revealed significant differences in SUAS and SCL-90-R scores between the pre-COVID-19 and post-COVID-19 groups, as well as among subgroups of inmates with suicide risk (Blaauw+) and high suicidal intent (SUAS+). The results of the non-parametric analysis are presented in Table 2. Significant differences were observed in most SUAS and SCL-90-R scores. However, specific items, such as resourcefulness, inability to feel emotions, tension, and somatic concerns, did not show significant differences. Inmates with high suicide risk and high suicidal intent admitted after COVID-19 reported significantly higher levels of psychological distress (GSI) and greater symptoms of impulsivity, interpersonal sensitivity, hostility, phobic anxiety, paranoid ideation, and psychoticism. They also exhibited a lower frustration tolerance.

### 3.4. Logistic Regression Analyses

Binary logistic regression analyses were conducted on data from inmates meeting the criteria for suicide risk (Blaauw+; N = 1347). The results, as presented in Table 3, are summarized below. Regression Model A: obsessive-compulsive, phobic anxiety, and paranoid ideation symptoms significantly contributed to the prediction of suicidal intent, correctly classifying 76% of high-intensity (SUAS+) and 75% of low-intensity (SUAS−) cases. Model B: inmates’ perceived loss of control and anergia were positively associated with the logit probability of suicidal intent, classifying 90% of high suicidal intent intensity and 83% of low intensity. Nagelkerke’s R^2^ = 0.712 indicates a strong explanatory value for this model. Model C: only having family of origin as the inmate’s social net explained 65% of the variation in obsessive-compulsive symptoms (*p* = 0.007; OR = 3.308). Model D: imprisonment after the onset of COVID-19 (*p* = 0.010; OR = 8.908) and substance abuse (*p* = 0.023; OR = 5.479) were positively associated with phobic anxiety, correctly classifying 92% of symptoms above the clinical attention threshold (PHOB > 65 T). Model E: female gender (*p* = 0.027; OR = 11.485) and post-COVID-19 incarceration (*p* = 0.047; OR = 3.718) were positively associated with paranoid ideation.

## 4. Discussion

The significant increase in suicide rates in prisons worldwide, particularly amidst the COVID-19 pandemic and in contrast to general population trends, prompts several important considerations. Prisons, as enclosed environments, pose unique stressors and challenges, including overcrowding, restricted movement, and forced cohabitation. The uncertainty surrounding the duration of the pandemic, its impact on legal proceedings, and the potential for outbreaks within prison may have contributed to heightened feelings of hopelessness among inmates. Pandemic-related restrictions, such as the replacement of in-person visits with video calls and reduced activities, have likely intensified social isolation. These factors could have intensified psychosocial vulnerabilities, emotional distress, and health issues among the incarcerated population. Our primary focus is on identifying individuals’ healthcare needs, taking into account the impact of changing circumstances, and implementing measures to prevent suicides in correctional facilities.

As part of the Regional Plan for the Prevention of Suicidal Behavior in the Adult Prison System, the U.O.S.D. Department of Adults and Minors Healthcare Criminal Area of the Local Health Authority (ASL) of Salerno implemented the SRSA protocol. The results of the protocol, carried out on a substantial sample of newly admitted inmates from 2017 to 2023, showed that approximately 64% of them met the criteria for suicide risk upon their arrival in prison. The majority (91%) of these individuals were Italians, predominantly aged between 40 and 59 years, as opposed to those without suicide risk. Additionally, only 7.3% of the at-risk group reported a high level of suicidal intent intensity. It is crucial to note that, in our sample, even though the percentage of new inmates evaluated as at risk of suicide over the past four years (59%) was lower than that of inmates incarcerated prior to the pandemic (70%), a statistically significant difference was found in the psychosocial profiles of these two groups. 

Following the outbreak of the coronavirus disease, we observed a higher proportion of inmates aged 60 and above at risk of suicide and with high suicidal intent. This finding aligns with overarching trends in the general population, where the elderly have emerged as a significantly affected group in the post-COVID-19 era. Our study spotlights the vulnerability of older inmates, echoing prior research [49], which identifies elderly individuals within the prison environment as being at an increased risk of suicide. Beyond categorizing the elderly as a high-risk group, previous results revealed them as a cohort marked by distinctive health issues, social-sensory disorientation, and an intensified sense of alienation [50]. Recent studies further support the negative impact of pandemic social distancing policies on the health of older individuals. These policies have led to increased isolation, prolonged stress, and the burden of other factors [51], which have been linked to an elevated suicide risk among inmates [27,52,53]. Recognizing the elderly as a majorly affected group not only deepens our understanding of the challenges faced by older inmates but also highlights the broader societal impact of COVID-19 on this demographic. During the pandemic, compared to the period before, a significantly higher percentage of newly admitted inmates with high suicidal intent lacked family or emotional support. Consistent with previous meta-analytic research that has linked the absence of social connections and low perceived social support to an increased risk of suicidal behavior in inmates [21,54], as well as higher levels of suicide ideation among those identified as high-risk for suicide [55].

This study found that 86% of inmates at risk of suicide, incarcerated after the onset of COVID-19, reported substance abuse problems, and 77% had a history of repeat offending. In comparison, for those at risk before the pandemic, the figures were 78% and 44%, respectively. An updated systematic review has also shown evidence of increasing substance use disorders in prison over the past three decades [56]. Considering the subsequent adverse outcomes, such as the risk of suicidal behavior, violent re-offending, and post-release mortality [16,57,58,59,60], targeted psychosocial treatments are clearly warranted [61,62]. Moreover, there was a significant increase in violent offenses against persons, rising from 9% before 2019 to 14% in the post-COVID-19 group and subgroup. These data align with the heightened risk of domestic violence associated with pandemic measures and substance use disorders [63]. Dysregulated behaviors, including interpersonal violence, self-injury, and substance abuse [64], appear to be linked to increased suicidal ideation [23]. Previous cluster analysis has outlined that inmates with dysregulated behavior—marked by severe impulsiveness; compromised emotional relationships; and a lack of family or social contacts during their custody—displayed greater psychological distress and a higher tendency towards suicidal behaviors [13].

In addition to the common vulnerabilities among individuals deprived of their liberty, inmates with a high level of suicidal intent intensity who entered prison after the pandemic onset reported significantly higher severity of psychological distress while also demonstrating lower frustration tolerance. Incarcerated individuals are more likely to suffer from anxiety, paranoia, psychosis, and suicidal behavior, which have been exacerbated by the pandemic [28,29,30,31,65,66,67]. Considering the positive association between anxiety, low self-control, and suicide attempts in prison [21,23], our findings suggest that, even after controlling for the severity of distress, perceived loss of control, anergia, obsessive-compulsive behaviors, phobic anxiety, and paranoid ideation are positively linked to suicidal intent. These results highlight the prevalence of mental and emotional vulnerability in correctional settings. Future research could yield valuable insights into how the Big Five Personality traits may intersect with the factors identified as associated with high suicidal intent among newly incarcerated individuals. Furthermore, when adjusting for the intensity of suicidal intent, we observed that having only a family of origin as a social network was positively associated with obsessive-compulsive symptoms characterized by persistent, ego-dystonic, and distressing thoughts or impulses. This may be due to the vulnerability factors associated with being single and not having children, which contribute to current and lifetime suicidal ideation in individuals troubled by obsessive thinking and compulsive behaviors [68,69]. On one hand, the association between paranoid ideation symptoms (such as hostility, suspiciousness, and delusions) and the intensity of suicidal intent experienced by female inmates may be due to heightened perceptions of harm and concerns about victimization [70], which are more pronounced within the specific context of women’s prison. On the other hand, substance abuse prior to incarceration showed a positive association with phobic anxiety symptoms (i.e., irrational fearful reactions to persons or situations), which were significantly linked to high suicidal intent. Notably, when controlling for high suicidal intent, imprisonment occurring after the onset of COVID-19 was positively associated with both paranoid ideation and phobic anxiety symptoms. 

The well-established co-occurrence between anxiety and substance use disorders can be explained by shared vulnerability factors, self-medication, and substance-induced models [71,72]. As newly admitted inmates with substance use problems may experience withdrawal and anxiety symptoms within the first hours and days of detention, it is crucial to carefully assess and monitor their suicide risk over time. The Multidisciplinary Addiction Team (MAT) of the U.O.S.D. Department of Adult and Minor Healthcare, Criminal Area of the Local Health Authority (ASL) of Salerno offers specialized treatment to inmates with pathological dependence within three local penitentiary institutions: CC Salerno, ICATT Eboli, and CR Vallo della Lucania. 

Overall, the findings of this study contribute to a more nuanced understanding of the pre-existing psychosocial vulnerabilities that inmates ‘import’ into prison and that, when compounded by stressful circumstances (such as COVID-19), may increase the likelihood of suicide attempts [20,21,22,23]. Interestingly, correctional physicians highlighted the tendency amongst prison staff to ‘psychiatrize’ behavioral difficulties, overestimating the presence of psychiatric disorders [73]. It is essential to distinguish between psychiatric illnesses, which tend to be enduring, and psychological distress, which is typically context-dependent and transient. Based on our experience, we believe that the perception of inadequate social support and reduced opportunities for occupational, educational, and recreational prison activities may lead inmates to employ maladaptive coping strategies, exacerbating the underlying pain of imprisonment. In this context, mental distress can have a detrimental effect on the well-being of both inmates and staff, and it can even strain inmate-staff relationships. This underscores the importance of promptly implementing interventions to alleviate discomfort and address contextual distress by enhancing coping skills among the inmate population and correctional staff, including prison police, pedagogical officers, and health professionals.

One limitation of the current study is the lack of information regarding the inmates’ family history of suicide and other psychiatric illnesses. Exploring these aspects in future research would contribute to a more comprehensive understanding of the factors influencing suicidal intent in prison. Another limitation is the absence of data on adverse childhood experiences and life events that may impact an inmate’s adaptation to the prison environment, potentially contributing to the development of suicidal ideation. We strongly advocate for future research to investigate the interplay between trauma-related factors, psychosocial vulnerabilities, and specific stressors associated with custody, all of which may increase the likelihood of suicidal behaviors among inmates. A prospective longitudinal study applying the SRSA protocol to the same inmates on their first day and at various points during their incarceration could provide more robust insights into the effectiveness of psychosocial factors linked to suicidal intent, as identified in this work. Such a study might also shed light on whether targeted interventions aimed at mitigating contextual distress can result in a reduced risk of suicide. 

Notwithstanding the mentioned limitations, our contribution holds value for health professionals, particularly those operating within correctional institutions, as it seeks to heighten awareness of suicide risk and its related factors.

## 5. Conclusions

The rising global prison suicide rates, especially during the COVID-19 pandemic, emphasize the crucial necessity for focused screening efforts and adaptability to evolving circumstances. Our comprehensive examination of factors associated with suicidal intent before and after the pandemic onset reveals the intricate interplay between inmate vulnerabilities and external stressors. Post-pandemic incarcerated individuals reported heightened suicidal intent linked to factors such as older age, limited social connections, substance abuse, recidivism, elevated psychological distress, and lower frustration tolerance. This underscores the urgency of a Suicide Risk Screening and Assessment (SRSA) protocol aimed at identifying high-risk inmates and guiding multi-professional interventions to address specific aspects of distress. Effectively managing suicide risk requires a thorough understanding of the individual’s current health state, coping strategies, social support, and their ability to adapt to the environment. Despite the complexity of motivations behind suicide attempts and unforeseen contingencies, psychosocial vulnerability screening upon prison admission, preventive interventions, and regular reassessment throughout incarceration are indispensable. In the post-pandemic phase, continuous monitoring and longitudinal studies are imperative to assess the impact of vulnerabilities and stressors on suicidal behavior. Ongoing research remains vital for refining suicide prevention strategies and tailoring them to the actual needs of inmates, aiming to reduce alarming suicide rates in prisons. Essentially, tackling the complex challenge of suicide prevention demands a multifaceted approach encompassing early identification, targeted interventions, and continuous evaluation to create a safer correctional environment.

## Figures and Tables

**Table 1:** Descriptive statistics of socio-demographic, clinical, and legal characteristics of inmates incarcerated pre-and post-COVID-19 pandemic.

	Total Sample (n = 2098)	Pre-COVID-19		Post-COVID-19		Test Statistic				
		Total (n = 1043)	Blaauw+ (n = 726)	Total (n = 1055)	Blaauw+ (n = 621)	Total		Blaauw+		
						*χ^2^*	*p*	*χ^2^*	*p*	*V*
Gender										
Males	1965 (93.7%)	983 (94.2%)	677 (93.3%)	982 (93.1%)	587 (94.5%)	1.203	0.273	0.940	0.332	0.03
Females	133 (6.3%)	60 (5.8%)	49 (6.7%)	73 (6.9%)	34 (5.5%)					
Age (range)										
18–39	1125 (53.6%)	567 (54.4%)	343 (47.2%)	558 (52.9%)	263 (42.4%)	0.457	0.499	3.239	0.072	0.05
40–59	843 (40.2%)	425 (40.7%)	344 (47.4%)	418 (39.6%)	303 (48.8%)	0.277	0.599	0.266	0.606	0.01
≥60	130 (6.2%)	51 (4.9%)	39 (5.4%)	79 (7.5%)	55 (8.9%)	6.092	0.014	6.261	0.012	0.07
Nationality										
Italian	1848 (88.1%)	872 (83.6%)	664 (91.6%)	976 (92.5%)	566 (91.1%)	39.746	0.000	0.083	0.773	0.01
Non-Italian	250 (11.9%)	171 (16.3%)	61 (8.4%)	79 (7.5%)	55 (8.9%)					
Education (years)										
0–11	1710 (81.5%)	881 (84.5%)	620 (85.4%)	829 (78.6%)	511 (82.3%)	12.070	0.001	2.409	0.121	0.04
12–18	388 (18.5%)	162 (15.5%)	106 (14.6%)	226 (21.4%)	110 (17.7%)					
Marital status										
Single	748 (35.7%)	377 (36.1%)	221 (30.4%)	371 (30.3%)	222 (35.8%)	0.205	0.651	4.360	0.037	0.06
Married/cohabitant	1081 (51.5%)	552 (52.9%)	422 (58.1%)	529 (57.9%)	301 (48.5%)	1.625	0.202	12.552	0.000	0.10
Separated/widowed	269 (12.8%)	114 (10.9%)	83 (11.4%)	155 (14.7%)	98 (15.8%)	6.640	0.100	5.441	0.020	0.06
Occupation										
Employed	1143 (54.5%)	626 (31.6%)	308 (42.4%)	517 (49.0%)	282 (45.4%)	25.658	0.000	1.213	0.271	0.03
Unemployed	955 (45.5%)	417 (40.0%)	418 (57.6%)	538 (51.0%)	339 (54.6%)					
Social net										
No family	353 (16.8%)	169 (16.2%)	75 (10.3%)	184 (17.4%)	120 (19.3%)	0.574	0.449	21.864	0.000	0.13
Family of origin	633 (30.2%)	336 (32.2%)	233 (32.1%)	297 (28.2%)	169 (27.2%)	4.110	0.043	3.806	0.051	0.05
Extended family	1112 (53%)	538 (51.6%)	418 (57.6%)	574 (54.4%)	332 (53.5%)	1.681	0.195	2.295	0.130	0.04
Previous self-harm										
Absence	1917 (91.4%)	947 (90.8%)	651 (89.7%)	970 (91.9%)	547 (88.1%)	0.876	0.349	0.855	0.355	0.02
Presence	181 (8.6%)	96 (9.2%)	75 (10.3%)	85 (8.1%)	74 (11.9%)					
Previous suicide attempts										
Absence	1937 (92.3%)	951 (91.2%)	647 (89.1%)	986 (93.5%)	561 (90.3%)	3.850	0.050	0.538	0.463	0.02
Presence	161 (7.7%)	92 (8.8%)	79 (10.9%)	69 (6.5%)	60 (9.7%)					
Substance addiction										
No substance abuse	411 (39.4%)	207 (19.8%)	159 (21.9%)	204 (19.3%)	85 (13.7%)	0.087	0.768	15.222	0.000	0.11
Substance abuse	1687 (80.4%)	836 (80.2%)	567 (78.1%)	851 (80.7%)	536 (86.3%)					
Offender										
First offence	775 (36.9%)	521 (49.95%)	408 (56.2%)	254 (24.1%)	142 (22.9%)	150.759	0.000	153.921	0.000	0.34
Recidivist	1323 (63.1%)	522 (50.05%)	318 (43.8%)	801 (75.9%)	479 (77.1%)					
Crime										
One type	1469 (70.0%)	795 (76.2%)	563 (77.5%)	674 (63.9%)	377 (60.7%)	38.022	0.000	45.013	0.000	0.18
Different types	629 (30.0%)	248 (23.8%)	163 (22.5%)	381 (36.1%)	244 (39.3%)					
Drug-related crime	1179 (56.2%)	619 (59.3%)	420 (57.9%)	560 (53.1%)	342 (55.1%)	8.369	0.004	1.052	0.305	0.03
Embezzlement	866 (41.3%)	397 (38.1%)	279 (38.4%)	469 (44.5%)	293 (47.2%)	8.840	0.003	10.494	0.001	0.09
Criminal conspiracy	487 (23.2%)	193 (18.5%)	139 (19.1%)	294 (27.9%)	172 (27.7%)	25.796	0.000	13.783	0.000	0.10
Violence against person	244 (11.6%)	97 (9.3%)	64 (8.8%)	147 (13.9%)	87 (14.0%)	10.956	0.001	9.073	0.003	0.08

Note: *χ^2^* = frequency analysis on demographic, clinical, and legal characteristics of two groups: pre-COVID-19 (n = 1043), post-COVID-19 (n = 1055), and of the two subgroups of inmates with suicide risk (Blaauw+; scores ≥ 24 on the Blaauw Scale) admitted before (n = 726) and after (n = 621) the onset of COVID-19; *p* = *p*-value was significant at 0.05 level; *V* = Cramer’s *V* effect size for chi-square test.

**Table 2:** Comparisons between suicidal intent and psychological distress measures in new inmates at risk of suicide.

	Pre-COVID-19		Post-COVID-19		Test Statistic			
	Blaauw+ (n = 726)	SUAS+ (n = 85)	Blaauw+ (n = 621)	SUAS+ (n = 13)	Blaauw+		SUAS+	
	M ± SD	M ± SD	M ± SD	M ± SD	U	*p*	U	*p*
Suicide Assessment Scale								
Sadness	2.08 ± 0.86	3.01 ± 0.75	1.61 ± 0.76	2.15 ± 1.07	156,528	0.000	285.0	0.002
Hostility	1.59 ± 0.80	2.29 ± 1.00	1.58 ± 0.87	2.62 ± 1.45	218,368	0.257	622.5	0.447
Anxiety	2.15 ± 0.84	3.18 ± 0.54	1.92 ± 0.83	2.85 ± 0.80	191,289	0.000	431.5	0.113
Low Self-esteem	1.39 ± 0.70	2.40 ± 0.98	1.20 ± 0.48	1.85 ± 0.90	197,051	0.000	384.0	0.065
Hopelessness	1.45 ± 0.74	2.59 ± 0.86	1.24 ± 0.52	1.85 ± 1.07	202,424	0.000	325.5	0.012
Tot. Affectivity	8.62 ± 2.77	3.47 ± 2.09	7.55 ± 1.90	11.31 ± 2.56	178,832	0.000	231.5	0.001
Resourcefulness	1.38 ± 0.71	2.59 ± 0.82	1.41 ± 0.67	2.62 ± 0.87	235,195	0.083	577.5	0.776
Perceived loss of control	1.45 ± 0.74	2.73 ± 0.81	1.29 ± 0.56	2.38 ± 0.77	205,220	0.000	426.0	0.141
Impulsivity	1.53 ± 0.82	2.24 ± 1.11	1.72 ± 0.90	3.00 ± 1.22	251,883	0.000	748.5	0.034
Poor frustration tolerance	1.81 ± 0.92	3.14 ± 0.64	1.67 ± 0.96	3.77 ± 1.17	203,355	0.001	781.0	0.005
Tot. Control and coping	6.16 ± 2.59	10.69 ± 2.11	6.09 ± 2.31	11.77 ± 1.74	229,661	0.538	733.5	0.055
Hypersensitivity	1.68 ± 0.83	2.64 ± 0.87	1.45 ± 0.73	3.15 ± 0.80	190,279	0.000	751.0	0.024
Emotional withdrawal	1.53 ± 0.79	2.76 ± 0.96	1.28 ± 0.62	2.62 ± 1.39	186,420	0.000	540.0	0.891
Inability to feel emotions	1.04 ± 0.42	1.11 ± 0.51	1.02 ± 0.21	1.38 ± 1.12	224,699	0.657	612.0	0.134
Tot. Emotional Reactivity	4.25 ± 1.56	6.51 ± 1.62	3.74 ± 1.22	7.15 ± 2.48	187,082	0.000	650.5	0.294
Anergia	1.49 ± 0.72	2.52 ± 0.85	1.23 ± 0.51	1.77 ± 1.17	182,522	0.000	282.0	0.003
Tension	1.71 ± 0.82	2.71 ± 0.84	1.66 ± 0.76	2.46 ± 0.97	220,213	0.424	478.0	0.378
Somatic concerns	1.37 ± 0.68	1.74 ± 0.87	1.33 ± 0.71	1.62 ± 1.19	216,413	0.092	469.0	0.332
Tot. Bodily states	4.57 ± 1.67	6.96 ± 1.64	4.22 ± 1.24	5.85 ± 1.28	206,607	0.006	313.5	0.011
Suicidal thoughts	1.12 ± 0.45	1.82 ± 0.99	1.03 ± 0.18	1.62 ± 0.65	214,095	0.000	519.0	0.704
Purpose of suicide	1.09 ± 0.37	1.67 ± 0.86	1.03 ± 0.19	1.46 ± 0.66	217,853	0.003	494.0	0.495
Wish to die	1.12 ± 0.44	1.86 ± 0.93	1.01 ± 0.10	1.15 ± 0.55	208,185	0.000	305.5	0.005
Lack of reason for living	1.11 ± 0.45	1.87 ± 1.01	1.01 ± 0.11	1.15 ± 0.55	211,677	0.000	316.5	0.007
Suicidal actions	1.05 ± 0.31	1.40 ± 0.82	1.00 ± 0.09	1.15 ± 0.55	219,935	0.000	472.5	0.239
Tot. Suicidal thoughts/ behaviour	5.48 ± 1.77	8.62 ± 3.84	5.08 ± 0.53	6.54 ± 2.70	210,408	0.000	365.5	0.045
Tot. SUAS	29.09 ± 8.34	46.27 ± 6.52	26.68 ± 5.11	42.62 ± 3.66	199,819	0.000	335.0	0.022
Symptom Checklist 90-R								
Somatization	48.89 ± 9.97	62.31 ± 11.91	46.53 ± 8.97	60.46 ± 8.90	190,564	0.000	482.5	0.458
Obsessive-Compulsive	45.91 ± 9.37	59.87 ± 11.14	43.12 ± 6.46	56.77 ± 9.67	189,614	0.000	466.0	0.362
Interpersonal Sensitivity	46.74 ± 9.15	59.93 ± 10.81	45.68 ± 7.55	68.15 ± 10.47	217,391	0.249	764.5	0.026
Depression	52.59 ± 11.40	70.04 ± 6.95	49.37 ± 9.31	71.38 ± 9.48	190,244	0.000	701.5	0.085
Anxiety	51.26 ± 11.26	67.22 ± 9.34	49.41 ± 9.27	71.15 ± 8.41	211,476	0.048	705.0	0.086
Hostility	44.51 ± 7.61	55.09 ± 11.64	45.06 ± 8.64	67.15 ± 11.02	227,443	0.765	871.0	0.001
Phobic Anxiety	48.24 ± 7.70	61.34 ± 11.91	48.64 ± 7.27	68.15 ± 10.63	239,249	0.025	748.0	0.036
Paranoid Ideation	46.24 ± 9.50	58.25 ± 11.70	44.27 ± 8.23	67.00 ± 13.34	196,993	0.000	801.5	0.009
Psychoticism	50.51 ± 9.52	64.02 ± 9.98	47.63 ± 8.00	70.15 ± 11.09	178,358	0.000	772.5	0.018
Global Severity Index	49.09 ± 10.80	66.92 ± 8.01	45.49 ± 9.67	70.69 ± 10.59	171,256	0.000	776.5	0.015
Positive Symptom Total	44.86 ± 8.80	59.96 ± 8.20	46.35 ± 9.84	68.69 ± 9.24	241,684	0.022	852.5	0.002
Positive Symptom Distress Index	54.38 ± 9.71	68.76 ± 7.85	51.62 ± 10.26	66.54 ± 8.84	180,364	0.000	443.5	0.234

Note: U = Mann–Whitney U test for independent groups applied to the Suicide Assessment Scale (SUAS item and total scores) and the Symptom Checklist 90-Revised (SCL-90-R dimensions and indexes T-scores) in subgroups of inmates with suicide risk (Blaauw+) and high suicidal intent (SUAS+), both incarcerated before and after the onset of COVID-19. The significance level (*p*-value) was set at 0.05.

**Table 3:** Statistics for binary logistic forward regression models on new inmates with suicide risk.

Variable in the Equation	B *	Wald (χ^2^)	*p*-Value	Exp(B) ^†^	95%Cl for Exp(B)	
					Lower	Upper
DV SUAS−/+ (Model A)						
IVs SCL-90-R						
Obsessive–Compulsive	0.068	6.991	0.008	1.070	1.018	1.125
Phobic Anxiety	0.075	13.000	0.0003	1.077	1.035	1.122
Paranoid Ideation	0.059	7.620	0.006	1.061	1.017	1.106
DV SUAS−/+ (Model B)						
IVs SUAS items						
Perceived loss of control	2.358	22.936	0.000	10.574	4.028	27.758
Anergia	1.635	12.016	0.000	2.127	5.129	12.929
DV OC−/+ (Model C)						
IVs socio-demographic						
Social net (only family of origin)	1.196	7.323	0.007	3.308	1.391	7.867
DV PHOB−/+ (Model D)						
IVs socio-demographic						
Post-COVID-19	2.187	6.709	0.010	8.908	1.703	46.612
Substance addiction	1.701	5.138	0.023	5.479	1.259	23.849
DV PAR−/+ (Model E)						
IVs socio-demographic						
Post-COVID-19	1.313	3.931	0.047	3.718	1.015	13.617
Female gender	2.441	4.902	0.027	11.485	1.323	99.684

Note: Forward logistic regression models were applied to inmates with suicide risk (Blaauw+: n = 1347). Model A: Suicide Assessment Scale (SUAS− vs. SUAS+) was the bimodal outcome, and Symptom Checklist 90-Revised T-scores served as predictors (controlled by GSI > 65 T); Model B: SUAS (−/+) was the bimodal outcome, and SUAS item scores were predictors (controlled by GSI); Models C, D, E: had SCL-90-R Obsessive-Compulsive (OC− vs. OC+), Phobic Anxiety (PHOB- vs. PHOB+), and Paranoid Ideation (PAR- vs. PAR+) as bimodal outcomes, with socio-demographic variables as predictors (adjusting for SUAS+). DV = dependent variable; IVs = independent variables; B * = regression coefficient; (χ^2^) = Wald-chi squared test; *p*-value = significant at 0.05 level; Exp(B) † = odds ratio; 95%Cl = 95% confidence.

## Data Availability

The data presented in this study are available on request from the corresponding author. The data are not publicly available due to privacy or ethical restrictions.
